# Guy’s Hospital Reports

**Published:** 1840-09

**Authors:** 


Art. III.—*Guy's Hospital Reports.* No. viii, April, 1839. Edited by George H. Barlow, M. A. &c., and James P. Barrington, M. A. 8vo. pp. 263. London, 1839.

The present number of this most excellent and highly practical work contains the following articles:—

On the Disorders of the Brain, connected with diseased Kidneys. By Thomas Addison, M. D.—On Perforations of the Stomach, from Poisoning and Disease. By Alfred T. Taylor.—On the Diurnal Variations of the Pulse. By William Augustus Guy, M. B., &c.—Observations on Poisoning by the Vapours of Burning Charcoal and Coals. By Golding Bird, M. D., &c.—Two cases of Poisoning by the Inhalation of Carburetted Hydrogen. By Thomas Pridgin Teale, F. L. S.—Case of Imperforate Uterus, with Remarks. By Alexander Tweedie.—On Incision in Cases of Occlusion and Rigidity of the Uterus. By Samuel Ashwell, M. D.—Observations on Fibrinous Concretions in the Heart. By Dr. Hughes, M. D., &c.—Analysis of Bones affected with Mollities Ossium. By G. O. Rees, M. D., &c.—Case of Division of the Tibia, for the Cure of Deformity occasioned by a Gun-shot Wound. By Charles Aston Key. Case of Spermatocele, or Varicocele, treated Excision of a portion of the Scrotum. By Bransby B. Cooper, F. R. S.—Observations on Abdominal Tumours and Intumescence: illustrated by Cases of Renal Disease. By R. Bright, M. D. F. R. S.

Our object is merely to present in a brief abstract the most interesting matter contained in the above enumerated articles.

### 1.—*On the Disorders of the Brain, connected with Diseased Kidneys. By Thomas Addison, M. D.*

In the first paragraph of his article, the author states the object of his communication to be:—

"First, To point out the general character and individual forms of cerebral disorder connected with interrupted function of the kidneys, from whatever cause such interrupted function may arise. Secondly, To shew, that, in recent as well as chronic disease of the kidney, the cerebral disorder is not unfrequently the most prominent, and occasionally the only obvious symptom present. And, Thirdly, To establish a means of diagnosis, in such obscure or in unsuspected cases, upon the peculiar character of the cerebral affection."

After stating the general character of cerebral disorder connected with disease of the kidneys, to be "marked by a pale face, a quiet pulse, a contracted or undilated and obedient pupil, and the absence of paralysis"—he enumerates the five following, as the individual forms:

"1. A more or less sudden attack of *quiet stupor;* which may be temporary and repeated; or permanent, ending in death.

"2. A sudden attack of a *peculiar modification of coma and stertor;* which may be temporary, or end in death.

"3. A sudden attack of *convulsions;* which may be temporary, or terminate in death.

"4. *A combination of the two latter;* consisting of a sudden attack of coma and stertor, accompanied by constant or intermitting convulsions.

"5. A state of *dullness of intellect, sluggishness of manner*, and *drowsiness*, often preceded by *giddiness, dimness of sight*, and *pain in the head;* proceeding either to *coma* alone, or to *coma accompanied by convulsions;* the coma presenting the peculiar character already alluded to.

"With respect to the first mentioned form of cerebral disorder connected with renal disease, that of quiet stupor, it is, in its most exquisite form, probably the least frequently met with; the face is pale, the pulse quiet, the pupil natural, or at least obedient to light; and although the patient may lie almost completely motionless, there is no paralysis; for, on attentively watching him for some time, he will be observed slightly to move all the extremities. By agitating him, and speaking loudly, he may sometimes be partially roused for a moment, but quickly relapses into stupor, as before; or it may not be possible to rouse him at all. There is little or no labour of respiration, no stertor, and no convulsions. Slight degrees of it, occasionally precede and pass into the next or second form.

"This second form of cerebral affection is that of a sudden attack of coma with stertor, or, in other words, apoplexy: it is, nevertheless, different from ordinary apoplexy: it is the serous apoplexy of authors, and presents the usual general characters of cerebral affection depending upon renal disease; for the face, instead of being flushed, is, in almost every instance, remarkably pale; the pulse, though sometimes small, and more rarely full, is remarkably quiet, or almost natural; the pupil, also, although occasionally dilated or contracted, is often remarkably natural in size, and obedient to light; and there is no paralysis. When the labour of respiration is very great, the general character is apt to be modified by an accelerated pulse, and occasionally by a slight flush of the countenance. The coma is for the most part complete, so that the patient cannot be roused to intelligence for a single moment. The stertor is very peculiar, and in a great measure characteristic of this form of cerebral affection connected with renal disease; it has not, by any means, in general, the deep, rough, guttural, or nasal sound of ordinary apoplexy: it is sometimes slightly of this kind; but much more commonly the stertor presents more of a hissing character, as if produced by the the air, both in inspiration and in expiration, striking against the hard palate or even against the lips of the patient, rather than against the velum and throat, as in ordinary apoplectic stertor: the act of respiration, too, is usually, from the first, much more hurried than is observed in the coma of ordinary apoplexy. The peculiar stertor coupled with the pale face has, in more instances than one, enabled me to pronounce with confidence the disease to be renal, without asking a single question, and in cases, too, in whcih no renal disease whatever had for a moment been suspected.

"The third form of cerebral disorder connected with renal disease is that of a sudden attack of convulsions. In this case, also, the countenance is, for the most part, remarkably pale, although, occasionally, slightly flushed at intervals: the pupil is often but little affected: in slight attacks of the kind, the pulse is sometimes singularly quiet; but when the convulsions are severe, and especially when there is such a degree of coma as to be attended with stertor, the heart often sympathizes, and the pulse becomes rapid, irregular, and jerking. This form of cerebral affection often passes into the fourth variety; or the cerebral affection shall take on the form of the fourth variety from the commencement: in the latter case, we have merely a combination of the second and third varieties—the coma, hurried breathing, stertor, and convulsions being so blended together, as often to have led to a dispute, whether the affection ought to be designated apoplexy or epilepsy. From what has been already stated, it may in general be very easily recognized as one of the common forms of cerebral disorder connected with renal disease.

"The fifth variety is that in which the cerebral disorder makes its approach in a more gradual and insidious manner, usually commencing with dulness of intellect, sluggishness of manner, and drowsiness, gradually proceeding to coma, and more or less stertor, with or without convulsions; these states being, at the same time, distinguished by the general indications already pointed out. This form of cerebral disorder appears to be that which most commonly supervenes in the progress of the morbid change of kidney described by Dr. Bright; and is very frequently preceded by giddiness, dimness of sight, and pain in the head."

After having thus given the symptoms and characteristics of cerebral disorder connected with renal disease, the Doctor devotes the remainder of his article to the consideration of a question essentially connected with and naturally growing out of the subject,—viz: "Whether there really exists any discoverable relation between the character of the renal affection and that of the brain—whether the form, permanence and violence of the cerebral disorder bear any relation whatever to the activity, duration, and extent of the renal disease." Although he confesses himself unable to come to any definite and decided conclusion upon the subject, yet he takes the affirmative of the question with some confidence of its truth, and urges in support of it—" a tendency to a state of quiet stupor," which varies from " sluggishness of intellect, to complete insensibility to surrounding objects," and which is observable in the milder and more transient affections of the kidneys. In connection with this, he gives a case in which these cerebral symptoms were strongly marked, but in which there were none of the ordinary symptoms of nephritis. Upon a post mortem examination, "the cortical parts of the kidneys were found highly injected, of a deep red, or almost chocolate color, and somewhat softened in its texture." He again urges the peculiar convulsions, already described, sometimes proving fatal; from which in other cases the patient recovers, and which he has observed "most frequently in renal dropsy, subsequent to scarlatina,"—" and in that form of renal dropsy," which proceeds from cold, and is commonly termed idiopathic. Lastly, he urges the cerebral affection, and " peculiar liability to be suddenly seized with the most alarming and most fatal of all the forms of cerebral disorder occurring in connection with renal disease—profound coma and stertor, with or without convulsions," which are *sometimes* observed in those cases of granular degeneration of the kidneys, as described by Dr. Bright. In regard to this last, he confesses that many cases occur unattended by any cerebral symptoms. All of the facts adduced by the author may be coincidences, or they may not: and they require additional observation to establish their necessary connection.

### 2.—*Perforations of the Stomach from Disease and Poisoning. By Alfred S. Taylor.*

This paper of Mr. Taylor's relates to the subject as connected with some questions in legal medicine, and his object is to point out the means of diagnosis in cases of death from, or attended with perforations of the stomach, in which poisoning had been or may be suspected.

In treating his subject, (which he does in a very perspicuous and satisfactory manner,) he considers separately the different causes of perforation—1st. perforation from poisons. 2nd. perforation from morbid causes. 3rd. perforation by solution.

In regard to the first he says: "Poisons are capable of inducing perforation of the stomach in two ways: 1. by corrosion—2. by leading to ulceration, and the destruction of the parietes in a circumscribed space by that process." Perforation by corrosion is decidedly the most common, in this division of its causes: and is effected by the "concentrated mineral acids and alkalies, corrosive sublimate, nitrate of silver, and a few others"—the action of which is "purely chemical." The corrosive poisons when taken, immediately produce very severe symptoms, (which the author does not give,) such as those of the most violent gastritis; with excruciating pain in the stomach, severe burning in the throat, &c., most commonly terminating speedily in death. Taking sulphuric acid as an example, (as it is the one most commonly used,) the above symptoms are produced in a very aggravated form; and the post-mortem appearances presented, will be extensive corrosion of the mouth, fauces, and œsophagus; and a perforation of the stomach, sometimes of considerable size, having "rough, irregular edges, often softened and pulpy." The mucous membrane for some space around the perforation presents a dark brown or black color, of a "sooty appearance." The black matter being removed from the surface, traces of inflammation will be found, provided the individual has lived long enough for that process to have been established. Besides these, the acid passes through the perforation which it has made in the stomach, and extends its ravages to the contiguous viscera. Its presence may be detected in the fluids contained in the stomach, or the cavity of the peritoneum, or in the corroded mucous membrane. Perforation may be produced by nitric acid also; but not so many cases of it have been observed, as it is not so commonly employed as the sulphuric. In those however which have occurred, the edge of the aperture is "generally tinged of a yellow color; or if bile be present in the contents of the stomach, then the mucous coat may have a greenish hue." Perforation has sometimes, but rarely, been produced by oxalic acid, some of the alkalies, particularly potassa, bichloride of mercury, nitrate of silver, and sulphate of copper.

In regard to perforation from ulceration and sloughing as the immediate cause, it is produced by what the author calls "the irritant poisons," which indeed is a generic division of Christison, including the corrosive poisons above spoken of. The author regards perforation from this cause as extremely rare, but that it has been produced probably two or three times by arsenic, and possibly one or two other substances. The perforation when it does occur, very much resembles that from previous disease, and requires the concurrence of a number of collateral circumstances to establish the fact, that it has been produced by poisoning.

With respect to the second class of causes of perforation, viz: "morbid causes," he says it may be a result—"1. of simple ulceration, sometimes of an acute, but more frequently of a chronic character. 2. of scirrhous ulceration." The first is intended to designate an aperture in which there is an ulcer of some size, of the mucous coat, having well defined but not elevated edges—an ulceration of less dimensions of the muscular coat, and one of still less of the peritoneal coat; thus forming a funnel-shaped perforation; the apex of the funnel being at the peritoneal surface. The second is intended to designate one of the same kind, save that the border of the ulcer and coats of the stomach contiguous to it are elevated, and so hard as to seem "almost cartilaginous."

A person laboring under the first form of ulceration, will suffer from some slight dyspeptic symptoms, or some trivial derangement of the stomach which attracts but little attention, when suddenly, (most commonly after eating,) they will be attacked with vomiting and severe and even excrutiating pain in some part of the abdomen, which continues despite all the means which may be employed, and the patient succumbs in, generally, from eighteen to thirty-six hours. Vomiting though commonly an attendant, is sometimes absent; there is no diarrhœa, the bowels being in general obstinately constipated; and pain between the shoulders has been so frequently observed in these cases as, in the author's estimation, to merit attention, as being probably something more than a mere coincidence. According to the author's observation and the cases which he has introduced into his article, this disease "seems to attack frequently young females, from 18 to 23 years of age, generally unmarried," though it is by no means exclusively confined to them. The post-mortem appearances present, in addition to the ulceration above described, and which is commonly from half an inch to an inch in diameter, and "almost constantly situated in or near the lesser curvature, between the cardia and pylorus;" "all the marks of severe peritonitis; effusion of serum with coagulable lymph; agglutination of the viscera, and extravasation of the contents of the stomach."

The symptoms of scirrhous ulceration differ from those of the simple form, in there having been for a long time gastric symptoms, such as irritation, &c., together with constitutional disturbance. But when the perforation takes place, and the fatal attack of peritonitis comes on, the symptoms are the same as those above detailed; and the post-mortem appearances are the same, save in the character of the ulceration, which has been distinguised above. And in addition to the characteristics of the scirrhous ulcer already given, we may add that the coats of the stomach are thinned off from within outwards, the peritoneal being reduced to a sharp smooth edge, without any appearance of laceration, as if it had been effected by gradual absorption.

By "perforation by solution" is meant a large opening found in the stomach, generally at its greater extremity, and occupying a large portion of the fundus. "It is large and irregular; the edges are thin, ragged, commonly much softened for a considerable space around, and present that fringed appearance which the stomach might be conceived to acquire by the scraping of its parietes with a blunt knife. "Most commonly there is no vascularity nor trace of any other lesion of the stomach; and so also there is no trace of peritoneal inflammation, when extravasation of the contents of the stomach has taken place, which however does not always occur. 'Tis true that vascularity of the mucous membrane of the stomach may sometimes be present, but it is to be considered as incidental, and depending upon some other cause. In regard to symptoms the author says: "this singular change is either not indicated by any symptoms during life, or they are of so slight a kind as to lead to no suspicion of the mischief which is going on." The author devotes several pages to the description of cases which he has collected—to the consideration of the cause of the perforation, whether it be the result of disease, and takes place during life, or whether the change is purely post-mortem, effected by the solvent power of the gastric secretion on dead animal matter—and "whether the gastric secretion acts on the stomach by its vital or chemical properties." But we must be content to give only the "conclusions" to which he considers himself justified in coming. They are the following:

"1. That perforations of the stomach, from solution, are very rare in the human subject.

2. That they may occur in healthy and diseased states of the body.

3. That the perforation takes place after death; and depends on the action of the gastric secretion, which, in the opinion of some, is facilitated by a diseased state of the parietes of the stomach. But that the secretion is the chief, if not the sole cause, seems probable, from the fact, that the liver, spleen and diaphragm have also been found softened, the latter even perforated where lying near the aperture in the stomach. We must then imagine, either that these accidentally contiguous parts partake of the same disease as the stomach, or that disease of that organ is not necessary for its coats to become destroyed by the secretion.

4. That the secretion cannot be the healthy gastric-juice, but some altered state of that liquid. This is rendered probable by the facts—1. If it were the ordinary secretion, perforation would be much more common in healthy persons, dying suddenly, soon after a meal. 2. It would not be met with in diseased subjects, or those laboring under disease of the stomach.

5. That the exact nature of the liquid producing the change, and the circumstances to which it owes its solvent power, are unknown."

We come next to speak of the means of diagnosis, in all cases of perforation of the stomach, in reference to the medico-legal questions growing out of them. From what is said upon the subject we may glean the following:

1. That it is hardly possible to confound perforation from either "simple" or "scirrhous ulceration" with that produced by the corrosive poisons. For even in the absence of all general evidence in regard to some such subtance having been taken, a simple inspection of the mouth, faucus, œsophagus, the perforation itself, and perhaps the corrosion of the surrounding viscera, together with an examination of the matters vomited, "consisting of shreds of membrane and altered blood," mixed with sulphuric acid or whatever may have been taken—and the contents of the stomach and cavity of the peritoneum—will settle the question almost beyond the possibility of doubt.

2. That it is principally in distinguishing perforation by disease, from that produced by the "irritant poisons" the difficulty consists. In order to do this with any degree of certainty, it is necessary to take a good many circumstances into consideration.

1. Perforation by disease is comparatively frequent, and moreover seems most apt to attack young females, while that by arsenic, (as an example of the irritant poisons, and the one most commonly employed,) is very rare.

2. In the former, the symptoms do not generally make their appearance for several hours after eating, though occasionally in much less time; whereas those from arsenic almost uniformly come on in about half an hour after the substance containing it has been taken.

3. In cases of the former, the pain in the abdomen comes on suddenly, is intensely severe, and may be felt in any part of the abdomen; while that from arsenic is one of a burning character, comes on gradually, increases slowly, and is confined to the region of the stomach.

4. "In perforation, vomiting, if it exists, is commonly slight; and it is chiefly confined to what is swallowed. There is no purging; the bowels are generally constipated. In arsenical poisoning, the vomiting is usually severe, and diarrhœa is seldom wanting."

5. In the former death takes place in from eighteen to thirty-six hours, and very often within twenty-four; whereas in the only case given by the author in which perforation was produced by arsenic, the individual lived four days; and of the great number of reported cases in which death resulted from arsenic in twenty-four hours, perforation was not found in one. Indeed it can hardly be supposed that arsenic could effect perforation in so short a space, as it has to accomplish it through the medium of inflammation and ulceration.

6. "In perforation, peritonitis is the sole cause of death. In arsenical poisoning, the fatal result takes place under the peculiar symptoms produced by the poison."

7. In the former, the marks of severe peritoneal inflammation will be found; while in perforation from arsenic, these may also exist, but not necessarily, for the individual may die so soon after perforation has taken place, (from the other effects of the poison,) that there has not been sufficient time for the peritoneal inflammation to set up. Even though this should be found, there will also be the effects of the poison upon the mucous coat.

8. In perforation from disease, there is not necessarily any inflammation of the mucous membrane of the stomach and small bowels, although it may sometimes be present; while in that from arsenic, the inflammation of the stomach and duodenum, is, generally, considerable, and not unfrequently is also found in the fauces, œsophagus, and rectum.

9. In perforation from disease, no poison will be found, while in that from arsenic, we may expect to find it, either in the matters vomited, in the contents of the stomach, or in the substance of the viscera.

And lastly, if several persons have partaken of the same food, and but one of them is taken ill shortly after, it is strong presumptive evidence against the supposition of poisoning; while, if they are all taken suddenly ill, the reverse obtains.

In regard to perforation by solution; if any thing be known of the symptoms under which the patient labored, there will be but little difficulty in making out the diagnosis, for if the perforation be cadaveric, the disease by which death has been produced will have been characterized by its symptoms, whereas if it have been produced by poisoning, its very evident symptoms will have been present. In case however that none of these facts be attainable, there will be some danger of confounding it with perforation by the "corrosive poisons." The aperture itself simulates, in no inconsiderable degree, that produced by sulphuric acid, for instance; while the contiguous viscera have the appearance of having been acted on by some corrosive substance. But still a diagnosis may be made out with some certainty; by inspecting the condition of the fauces, œsophagus—and by applying chemical tests to the contents of the stomach, and cavity of the peritoneum, together with the coats of the stomach. And if the mucous membrane of the stomach and bowels, together with the peritoneum, evince marks of inflammation, it will greatly heighten the probability of poisoning, provided other circumstances concur to the same point.

The last ten pages of his paper Mr. Taylor devotes to the consideration of the following medico-legal propositions:

"1.—A person may have died from perforation of the stomach through disease and not from poison.

"2. A person laboring under the disease, may be the subject of poison.

"3.—A person laboring under the disease, may have received blows or injuries on the abdomen, in which case it may be necessary to state, whether the perforation did or did not result from the violence used.

"4.—A perforation of the stomach from post-mortem changes may be mistaken for perforation from poison."

In connection with the first, he gives a very interesting case, in which the circumstances of a moral nature—the symptoms under which the patient labored—and some of the post-mortem appearances, were very strongly in favor of the supposition of poisoning, but upon applying the diagnostic tests, already detailed, for perforation by ulceration, the case was made out so clearly that it was not even deemed necessary to have a coroner's inquest over the body.

Two questions very naturally grow out of the second proposition.—"1. Whether the perforation, or the diseased state of the stomach leading to it, was due to poison. 2. Whether the disease or poison was the cause of death."—"The first question will be answered by considering the nature of the substance. Thus, knowing that the corrosives and irritants alone are liable to cause perforation of the stomach, the discovery of a narcotic poison will shew that the disease and the substance taken, could not have had any connection with each other. So again among the irritants, there are some, perhaps the greater number, not likely to be followed by perforation of the stomach."—The second may be determined, by ascertaining the symptoms under which the patient labored; whether those of ulceration, or of poisoning and by the appearances found upon dissection. See the characteristics of perforation by ulceration, and those by poisoning, already detailed.

With respect to the third proposition, any case of the kind may be determined, with some degree of certainty, by considering the nature of the violence inflicted, and the character of the perforation. Thus, if it proceed from the disease alone, a perforation peculiar to ulceration will be found; whereas, if from the violence, some laceration might be expected.

The fourth proposition presents a case which may possibly happen by the concurrence of extraordinary circumstances; but must necessarily be very rare. A case however may occur, in which the person may labor under the symptoms of irritant poisoning, and perforation be caused after death by the gastric secretion. In such a case, it will be necessary to take into consideration the general circumstances—to ascertain minutely the character of the symptoms—and attend to the post-mortem appearances, as already directed in such cases.

We have thus given an abstract of this paper of Mr. Taylor's, and trust that we will not be considered to have devoted more space to it, than was due to its merits.

3.—*On the diurnal variations of the Pulse. By William Augustus Guy, M. B. &c.*

In former numbers of these reports, Dr. Guy gave the results of his experiments on the effect produced by change of posture on the pulse of healthy males—and on the effect produced by the same cause, as modified by age and sex. The object of his paper now before us, besides that set forth by its title is to show the susceptibility of the pulse to effects by different agents, at different periods of the day.

The experiments are reported fully, and with minuteness; but as it is only desirable to show the general effects, it will suffice to notice the mean results.

Twenty observations made on himself, upon rising in the morning, and the same number, upon going to bed, gave as an average, sixty-four beats in the morning, and fifty-four at night. There was however between the highest number in the morning and the lowest at night, a difference of eighteen beats. "This remarkable diminution towards night" he observes, "took place in spite of the various excitements produced by food, study, or exercise, during a space of fifteen or sixteen hours."

His next series of observations was designed to show the state of the pulse throughout the entire day, in which it was counted every quarter of an hour, from nine and a half in the morning to twelve and a quarter at night: during which time he was engaged in a study, which produced no excitement of mind and required no change of position. From the results, it appears that there was a sudden increase in the number of beats, at a quarter to ten, from sixty to seventy-nine, produced by breakfast. That from this time there was a gradual diminution, until it got as low as fifty-two at quarter past four—that it remained at this, one hour and a quarter, when it suddenly increased again to seventy; which increase was produced by dinner. From this time there was an irregular diminution, falling and then rising again two or three beats, when, at a quarter to twelve, it was again at fifty-two. At twelve, and a quarter past twelve, it was at fifty-six and fifty-five.

Another single series of observations, from nine A. M., until two P. M., showed that the diminution towards evening is not constant; and also, that there may be considerable fluctuation in the morning as well as in the evening.

Another series designed to ascertain the effect of fasting upon the pulse, and which was protracted during a space of thirteen hours, showed a diminution of six beats, with "occasional fluctuations."

The next observations were made to institute a more exact comparison between the state of the pulse in the morning and evening, by examining it in the morning and evening under precisely the same circumstances. A set of observations consisting of eleven series was first made, to try the effect of continued rest on the morning and evening pulse. A second set, consisting of fourteen series was made, to try the effect of food on the same.

In regard to the first, the plan adopted was to ascertain its frequency before breakfast, as a standard of comparison throughout the day. Breakfast was then taken; and waiting until the pulse had subsided, from the increased frequency produced by the meal, to what it was before breakfast, it was counted every quarter of an hour, for a period varying in different series, from half an hour to two hours; and the mean frequency of the whole was noted. The minimum frequency was also noted. Remaining after dinner then in a state of rest, until the pulse had acquired the same frequency which it had before breakfast, (which was generally about eight or nine in the evening,) tea was taken, consisting of exactly the same with the breakfast, and the observations made as in the morning. The results also were noted as before. This was repeated eleven days, and a table constructed, containing the results of each day. The following is given by Dr. Guy, as "the mean results of the series given in the table."

| | max. | min. | mean |
|---|---|---|---|
| "Frequency of the pulse before the observations . . . . | 66 | 60 | 62.36 |

| | Morning: max. | Morning: min. | Morning: mean | Evening: max. | Evening: min. | Evening: mean |
|---|---|---|---|---|---|---|
| Average frequency of the several series of observations | 64.5 | 57.33 | 61.28 | 63 | 56 | 58.71 |
| Least number of beats in any series of observations | 62 | 56 | 59.18 | 62 | 54 | 56.54 |
| Differ. between the original frequency and least number | 6 | 0 | 3.23 | 8 | 4 | 5.82 |

In the second set of observations, viz: those to show the effect of food, precisely the same plan was adopted as in the above, and the fourteen series of observations give the following mean results:

| | max. | min. | mean |
|---|---|---|---|
| "Frequency before the meal . . . . . . | 66 | 60 | 62.08 |

| | Morning: max. | Morning: min. | Morning: mean | Evening: max. | Evening: min. | Evening: mean |
|---|---|---|---|---|---|---|
| Frequency after meal . . . | 81 | 70 | 75 | 74 | 60 | 69.15 |
| Increase . . . . . . . . . | 21 | 10 | 12.92 | 12 | 0 | 7.15 |
| Duration of effect in hours, | 3.75 | 1 | 2.02 | 1.75 | 0 | .09 |
| Diminution per hour . . . | 12 | 4 | 6.4 | 40 | 6 | 7.8 |
| *Exceptions to the rule of progressive diminution | 29.21 per cent. | | | 13.33 per cent. | | |

The following are the average results of six experiments, in which the pulse before the meal was sixty.

| | Morning: max. | Morning: min. | Morning: mean | Evening: max. | Evening: min. | Evening: mean |
|---|---|---|---|---|---|---|
| Frequency after the meal, . . | 81 | 70 | 75 | 72 | 60 | 63 |
| Increase . . . . . . . . . | 21 | 10 | 15 | 12 | 0 | 8 |
| Duration of effect in hours, . | 3.75 | 2 | 2.75 | 1.75 | 0 | 87 |
| Diminution per hour . . . | 6.9 | 4 | 5.45 | 20 | 6.8 | 9.02 |
| Exceptions to the rule of progressive diminution | 26.47 per cent. | | | 11.76 per cent. | | |

After noticing the uniformity of the results, the author remarks that, " by far the most remarkable fact established by these observations, is, that the same food which in the morning increases the frequency of the pulse from five to twelve beats, and keeps it raised above its natural number from one to two hours, may in the evening produce no effect whatever." He

*That is to say, the instances in which the pulse, instead of being less frequent, was more frequent than in the preceding observation, were in the morning in the proportion of 29 per cent., and in the evening of 13 per cent.

refers to his table of details, which our space does not permit us to insert.

Having given the results of his observations much more minutely and in extenso than we have noticed, he proposes the following as facts established by them:

"1. The pulse of a healthy adult male in a state of rest, unexcited either by food or exercise, is most frequent in the morning, and gradually diminishes as the the day advances.
2. The pulse diminishes in frequency more rapidly in the evening than in the morning.
3. The diminution of the frequency of the pulse is more regular and progressive in the evening than in the morning.
4. The effect of food is greater and more lasting in the morning than in the evening; and, in some instances, the same food which in the morning produces an effect considerable both in amount and in duration, has no effect whatever in the evening."

The theory which Dr. Guy proposes upon the subject, and in support of which he brings forward some additional facts and arguments, is that:

"Early in the morning, after we have been completely refreshed by sleep, the strength of the body is at its maximum; all its functions are performed with vigour; and all the causes of excitement produce their greatest effect. But activity of body or of mind, and the application of stimuli of different kinds, produce debility, which goes on increasing as the day advances. So long as the body continues in perfect health, this increasing debility produces a sedative effect on the action of the heart; the frequency of the pulse is diminished; and it becomes less and less affected by the application of stimuli. Sleep restores strength and vigour to the body, and again confers upon it that excitability of which fatigue had deprived it. But fatigue, or the want of rest, may give rise to a state of debility which is incompatible with health; and which is accompanied by increased frequency of pulse, and increased susceptibility of the action of stimuli. It is, of course, quite impossible to draw the line between that debility which is consistent with health, and that which in itself may be regarded as a departure from it— between that degree of exhaustion which is attended by torpor of the functions, and that which is accompanied by increased excitability: but it may be stated, as a general rule, that debility without disease is productive of an infrequent pulse."

Lastly, he suggests as a practical application, that in cases where we wish to afford nourishment with as little excitement as possible, we should give the food in the evening. And that where it is desirable to produce as great an effect as possible, by the exhibition of remedies, we should choose the morning.

4.—*Observations on Poisoning, by the Vapours of Burning Charcoal and Coals. By Golding Bird, M. D., &c.*

Passing over several pages of this paper of Dr. Bird's in which he institutes the question of—what is the poisonous ingredient in the vapours of burning charcoal, &c.—and in reference to which, from the facts and experiments adduced by him, he concludes that it is carbonic acid in a large majority of cases, but that in some few, some other unknown substance is probably the noxious agent,—we shall at once enter upon a notice of his "physiological and pathological effects resulting from the inhalation of charcoal vapour."

The "symptoms" produced by carbonic acid are well marked, but not entirely characteristic, as they simulate very nearly the premonitory symptoms of apoplexy. Under this head however, it is not desirable to condense the page in which the author portrays them, and we shall quote him at some length:

"The person exposed experiences an intense, penetrating, and throbbing headache, accompanied by a sense of weight and heat, especially about the occipital region; strong pulsation in and a sense of tightness across the temples; vertigo; increased action of the heart, often accompanied by violent palpitations; confusion of ideas, and partial failure of memory, accompanied, in many instances, by a disposition to nausea and hysteric sobbing. If the individual who has been thus exposed be now removed from the vitiated atmosphere, and placed in a current of cool air, especially in the recumbent position, with the application of hot water bottles to his feet, if they be (as under these circumstances they usually become) cold, these symptoms gradually vanish, and in a few hours the patient recovers. But if the person be not so fortunate, and remain exposed to the poisoned atmosphere, a buzzing noise in the ears succeeds, followed or accompanied by partial or total loss of vision; and an undefined, vague, feeling of intense horror or dread, which is rapidly succeeded by an irresistible disposition to somnolency or syncope. Subsequently, and in a space of time varying probably with the state of vitiation of the atmostphere or temperament of the individual, all power of volition disappears, the pulse and palpitation of the heart, which were previously above 100 or 200, diminish in frequency, falling to 40 or 50; respiration becomes slow and laborious; the surface universally cold, and often livid; the lips becoming blue or violet; the eyes retaining, in most cases, their lustre: gradually, these symptoms increase in intensity, with frequently the accession of tetanic convulsions, and in a few instances raging delirium, white or bloody foam appears before the mouth and nostrils, vomiting supervenes, and the sufferer expires in the act; occasionally, however, breathing his last without its occurrence; in which case, the tongue is found protruded, or clenched firmly between the teeth;—the countenance always retaining an expression of deep and placid calm, the victim being generally found lying on the fatal apartment in a calm and sleeplike attitude, which even the vomiting, that often occurs in the last moments of existence, had been as ineffectual in disturbing, as it had been impotent in arousing the sufferer to a sense of his perilous situation. These symptoms are, with a single exception, recognized by all toxicological writers as constant. The exception to which I allude, is, vomiting—a certainly not very unfrequent accompaniment of death by charcoal fumes, although I am aware it has been lately denied as almost impossible to occur. It is for us, however, to regard facts that have occurred, and therefore likely to happen again; rather than assertions of what ought, on theoretical grounds, to take place."

Having detailed the symptoms, the author devotes some space to an enumeration of the "appearances presented by the corpse"—and the "appearances on dissection." In regard to the former, he gives a table, containing the appearances observed in a number of cases: but the following extract will present the principal ones, and those most uniformly found:

"The body is almost invariably found sprinkled with livid spots of a blueish or reddish brown, passing into violet: these are most numerous in the most depending parts, as about the back and loins; frequently occurring on the extremities, especially on the fore-arm: the limbs are in some cases extremely flexible, in others as rigid; so that no general law will apply to these circumstances: the fingers are often irregularly bent, sometimes stiff and extended; the arms being sometimes thrown across the chest, especially if tentanic spasms have preceded death. The animal heat has been stated by many authors—as Henke, Jarger, Orfila, and Beck—to remain for a preternatural length of time; whilst numerous cases are on record in which the bodies cooled as rapidly as under ordinary circumstances. The same difference of opinion, among authorities of equal weight, exist on the rapid or slow development of the appearances of decomposition. The tongue is found projecting, and often firmly clenched between the teeth; unless vomiting preceded death, in which case the tongue is usually drawn in and concealed. The mouth is often covered with white or bloody foam; the face in some cases is red and bloated, in others pale and unswollen. The eyes generally retain their vivacious and lustrous aspect, rarely being dull; sometimes they are injected: the pupils have, in almost every recorded case, been found dilated;—the whole features presenting an appearance of calm repose; even slight distortion being extremely unfrequent. On examining the interior of the nostrils, they have been, in many cases, found lined with a black fuliginous deposit. The abdomen is generally found distended with air."

"*Appearances on Dissection.*"

Under this head also, the author gives a table, containg the pathological appearances found in 18 cases. The most common are—

*Head*—Its coverings injected with blood; internally, the membranes and sinuses "invariably turgid with blood," and serous effusion beneath the arachnoid. Cerebral mass—its surface injected, presents bloody points on being cut, and occasionally softened. The lateral ventricles contain serous effusion, sometimes very abundant. Surface of cerebellum turgid, and serous effusion at the base. Extravasation of blood sometimes takes place from ruptured vessels; and the author gives one case in which there was universal effusion between the arachnoid and pia-mater, dipping down between the convolutions. Blood in the brain sometimes black, at others florid.

*Chest*—Frequent effusion of bloody serum into the pleura; lungs sometimes expanded and full of blood and air, at others collapsed—vary in appearance from a perfectly natural one, to redness with black spots, and even to a blackish violet color; vessels sometimes empty, at others turgid; mucous membrane of air passages, generally reddish and frothy, sometimes pale, with florid or black blood.

*Heart*—frequently effusion of bloody serum into the pericardium, both ventricles sometimes empty, the right commonly filled; the left usually empty, but occasionally both are filled with florid or black blood.

*Abdomen*—Most commonly all the viscera are healthy; sometimes the mucous membrane of the stomach and duodenum has a reddish tint, but which is probably produced by a recent digestion; serous effusion into the peritoneum not unfrequent; arteries empty, veins turgid with dark blood.

The author next raises the question whether carbonic acid acts simply by excluding atmospheric air, or as a specific poison. From the symptoms, vertigo, headache, loss of vision, somnolency, &c., which make their appearance very soon after exposure to the gas, and before any pulmonary symptoms occur, and from some experiments, in which a gentleman immersed himself (and afterwards some birds) in this gas, and soon experienced its pecular effects, notwithstanding he breathed pure air by means of a tube; together also with the facts that animals exposed to a mixture of carbonic acid and oxygen expire suddenly, notwithstanding that the oxygen is in as large a proportion as it exists in the atmosphere; from all these, we say, together with other facts already detailed under different heads, the author conceives himself justified in making the following deductions:

"1. That carbonic acid gas, sufficiently diluted with air (as in charcoal-vapor) does not act fatally, by closing the glottis spasmodically, or by excluding oxygen, but as a specific poison.

2. That such an atmosphere will produce death; although it may contain a sufficient amount of oxygen to support life *per se*, and to allow the arterialization of the blood to proceed: on which account no dependence can be placed on the dark or florid color of the blood, as arguments for or against poisoning by carbonic acid gas.

3. That such diluted carbonic acid acts most probably on the nervous system, primarily; and, secondarily, by no means essentially on the circulating fluid.

4. That the death of persons inhaling an atmosphere vitiated by carbonic acid is produced by the accession of apoplexy, often attended by serous effusion into the ventricles, or on the surface of the brain; and occasionally even by extravasation of blood from some cerebral vessel.

5. That no dependence can be placed on the bloated and red, or pale and contracted features; on the liquidity or coagulated state of the blood; on the injection or paleness of the mucous membrane of the intestinal tubes or air-pipes; or on the flexibility or rigidity of the limbs; as positive arguments for or against the action of carbonic acid as a cause of death, in individual cases, falling under medico-legal investigation."

5.—*Two cases of Poisoning by the Inhalation of Carburetted Hydrogen. By Thos. P. Teale, F. L. S. (Communicated by C. Aeton Key, Esq.)*

This article details the circumstances connected with two cases of fatal poisoning by carburetted hydrogen gas, which occurred in Leeds, England, December, 1838. The two individuals, an old woman sixty-nine years of age, corpulent and subject to attacks of paralysis, and another female aged twenty-two years, in good health, occupying two small rooms on the ground floor of Potter's Almshouse, were, during the day previous to the night on which they suffered its fatal effects, annoyed by the smell of gas, which escaped from a rupture in a pipe that passed in a few feet of their room. Between the rupture in the pipe and a sink in the floor of a pantry which opened into their sleeping room, there was a free communication for the gas by means of loose earth and rubbish that intervened; and in the evening an explosion of the gas took place in the pantry from a lighted candle being taken into it. After the explosion, by which probably the greater portion of the gas then in the room was consumed, no smell of it was perceived, and apprehending no further danger, the two individuals spoken of retired to the same bed about ten o'clock. About nine the next morning, the neighbors becoming alarmed at their rising so unusually late, broke into the room, immediately perceived a smell of coal gas, the old woman was found lying on her side, lifeless, cold, and the muscles rigid; the young woman was lying upon her face, was dead, but the trunk and extremities were still quite warm and flexible. The bed clothes were but little displaced, and there was no other indication of any struggling by either of them.

Morbid appearances presented by an examination of the body of the young woman about ten hours after death:

External surface generally very pale, save some mottled florid discolorations on the neck and back; features not distorted; pupils moderately dilated; muscles rigid; fingers flexed.

*Head*—Scalp contained some, but was not loaded with blood; interior membranes, their vessels and sinuses in a normal condition; substance of the brain firm, contained a "very fluid and rather florid blood" which oozed at numerous points when cut into; lateral ventricles contained about an ounce and a half of transparent serum. A small quantity of serum was collected in the spinal canal, but there was no observable lesion in any of its contents.

*Chest*—The muscles exposed in opening this cavity, together also with those of the abdomen, exhibited a very light and florid appearance. The tissues generally of this cavity and the abdomen were pallid, and exhibited a "remarkable absence of turgidity of the veins.

*Heart*—Its right side contained a small quantity of fluid blood, not so dark as venous blood generally; left ventricle firmly contracted and empty.

*Lungs*—Substance generally much less crepitant than natural. Mucous lining red.

*Abdomen*—Mucous membrane of stomach, "thick, opaque, and easily detached, and presented reddened streaks upon its folds—mucous membrane of the small intestines, generally red, but presented patches in which the redness was most intense. This vascularity was most considerable in the jejunum, next in the duodenum, and least in the ileum. Throughout the same surface also, were "multitudes of extremely minute ecchymoses." Liver—fluid blood oozed freely from it on being cut. Gall-bladder distended with a thin watery fluid. "The uterus contained a little sanguineous mucus, and its internal membrane was red." (We will here remark that the catamenial discharge had ceased about two days before her death.) "The left ovary contained a body about the size of a pea, having the appearance of clotted blood. The urinary bladder was flaccid, and contained about half an ounce of opaque white fluid: its mucous membrane was white."

Morbid appearances presented by examination of the old woman.

*External*—Same as in the former case.

*Head*—Its external the same as in the other. Interior—Sinuses of the dura-mater, contained a moderate quantity of very fluid blood. Considerable sub-arachnoid serous effusion, which was more abundant upon the upper surface of the hemispheres—and somewhat turbid in same places. It was so considerable as to penetrate the fissures between the convolutions, separating and giving them the appearance of being atrophied. The arachnoid and pia-mater were easily detached from the surface of the brain. The substance of the brain contained fluid blood of a florid hue, which escaped at numerous points of an incised surface. Lateral ventricles empty—small, and exhibited numerous very firm adhesions.

*Chest.*—Generally the same as in the former case. Heart—right side contained rather more, but not turgid with blood, of the same kind, together with a small fibrinous concretion—left side not so firmly contracted, and contained a quantity of the same partially changed blood, together with a small fibrinous concretion. Lungs—more crepitant than in former case, presented the mottled appearance of age, with the intervals too red—considerable blood and frothy fluid oozed upon incision—and the mucous lining was injected.

*Abdomen.*—Stomach normal—small intestines distended with gas that "burnt with a blue flame"—their mucous membrane presented the same vascularity and ecchymosed appearance as in the former case. "Liver, large and granular, fluid blood escaping freely from its cut surfaces." All the other viscera healthy.

A portion of the gas from the small intestines was analyzed, and found to contain:

| | |
|---|---|
| "Carbonic acid, . . . . . . . | 36 |
| Carburetted hydrogen, . . . . . | 45 |
| Nitrogen, . . . . . . . . | 19 |
| | 100 |

It is no unusual thing however to find combustible gas in the intestines of persons who die under a variety of circumstances.

In concluding, the author expresses in plain terms, his opinion that the gas, if respired unmixed with air, would speedily prove fatal, by producing asphyxia; and seems inclined to believe that, in the two cases just detailed, it acted somewhat in the same way; at any rate not as a poison. In this opinion he is supported by experiments which have been made upon animals with this gas, and by the notorious fact that colliers frequently breathe for a considerable time, an atmosphere strongly impregnated with it, without suffering any serious consequences. But on the other hand, from some experiments made upon himself by Sir H. Davy, it would seem to be decidedly a narcotic poison. And again, the absence in both of these cases of any considerable discoloration of the skin, *engorgement* with blood of the liver, spleen and kidneys, and turgid state of the pulmonary vessels, right auricle and ventricle, and of the venous system generally, all of which are most commonly found in asphyxiated persons, renders it probable that such was not the cause of their death, and heightens the probability of its having acted as a narcotic poison. We cannot urge the similarity of the morbid appearances found, to those produced by the narcotic poisons, for, according to the observations which have as yet been made upon the subject, they are so various that no reliance can be placed upon them. We may add however, that if the gas had acted simply by excluding the due proportion of oxygen, and thence as an asphyxiating one, most probably one of the individuals would have suffered some unpleasant sensation, that would have awakened them, and thus have prevented the fatal effects, or at least have caused some disturbance; whereas there was no indication of their having moved much: which we would readily expect, upon the supposition of their having respired a narcotic poison, producing stupor and insensibility.

### 6.—*Case of Imperforate Uterus. By Alexander Tweedie.*

In the fourth number (vol. ii, p. 258,) of these Reports, is given the early history of this case, together with an account of her first delivery, and the treatment then employed. With regard to that part of the history of this patient, it is sufficient for our present purpose to say: that she was a rather small woman, but had always enjoyed robust health—had menstruated regularly from her fourteenth year, up to the time of her marriage, which was in February, 1836, in her 23d year—that she never menstruated after she was married—that in November, 1836, she was admitted into Guy's Lying-in Charity—shortly afterwards had a slight sanguineous discharge from the vagina, which continued a day or two—that in about twenty-four or thirty-six hours from its cessation, she felt the early symptoms of approaching labor—that soon the pains became pretty severe, and continued so but ineffectual, for some hours—that a smooth globular body was discovered to descend into the vagina at each contraction of the uterus—that no os uteri could be found—that the vagina could be traced throughout its whole extent, and was ascertained to be reflected from this globular body just as it commonly is from the neck of the uterus—that during the absence of pains, this body could be distinctly felt to be the head of the child with the relaxed coats of the uterus intervening—that a point was discovered, which seemed to be thinner than the rest, and an incision was made through it antero-posteriorly, to the extent of an inch and a half—that the waters immediately escaped—that subsequent pains caused a considerable extension of the opening by laceration, extending from the reflexion of the vagina at the ileo-pubic junction on the right side, obliquely across towards the sacro-iliac symphysis of the left—that after a protracted labor and considerable exhaustion, the patient was delivered of an asphyxiated child, which however was resuscitated—and that finally she recovered without any very unfavorable symptoms, leaving a small opening at the top of the vagina, with several cicatrized lines radiating from it, and without any cervix uteri.

The present article gives an account of her second confinement, which took place in January, 1838. Her health after her first delivery, had been tolerably good—she had miscarried twice, once at the second month, and the second time at the third month of utero-gestation—and she menstrated regularly during the whole period of lactation, when not pregnant. In this, her second confinement, when she had been in labour 8 hours, the uterine contractions became "intensely powerful," and on examination, there was found at "the uterine extremity of the vagina, an irregular opening, which posteriorly and laterally seemed continuous almost with the vagina, but anteriorly was bounded by a strong, firm, unyielding, rigid edge, upon which at each pain the child's head was forcibly impelled." The opening was about "the area of a penny," and a cicatrized line was distinctly perceptible, running towards the left ilio-pubic junction. The uterine contractions having continued very powerful for twelve hours, without augmenting the size of the opening, it was determined to enlarge it; which was accordingly done to the extent of an inch with a probe-poined bistoury, after the manner of dividing a hernial stricture. Scarcely any blood followed the incision—the uterine contractions almost ceased for a time, and she felt faint. Upon the exhibition however of some weak brandy she revived, the contractions returned, and in less than an hour from the operation, she was delivered of a full grown child, which was asphyiated but restored after some difficulty. As in her former confinement she recovered without any difficulty—suffering only for a few days some tenderness upon pressure over the pubes—and some inconvenience from distention of the left breast by milk, there being in this case the singular coincidence of the imperforate state of the os uteri and absence of the neck of the uterus, with the want of a nipple upon the left breast.

In his remarks upon the probable explanation of this case, Mr. Tweedie observes:

"There being thus no cervix, it is evident that the glandular or follicular structure of the part cannot exist; but it does not therefore follow that there was no opening into the womb prior to impregnation. We believe there was an opening, but not surrounded by the glandular structure which naturally exists here: hence, when impregnation took place, the ordinary mucous secretion could not be found, to seal it up; and is it very unreasonable to imagine, that, under this malformation of parts, adhesive matter, instead of mucus, might have been poured forth, and thus, by adhesion, as pregnancy advanced, the orifice have become entirely obliterated?"

For the probability of such a state of things having existed in this case, he relies upon the facts of it, as already detailed: and in support of the possibility of such an adhesion's taking place, he quotes first: a case by Dr. A. S. Thomson, of a woman aged 65—who died of dry gangrene—in whom the uterus was found containing 8 quarts of dark brown fluid—its mucous membrane healthy—and the os uteri interiorly "as completely obliterated as if it had never existed"—and internally, or upon its vaginal face, but "faintly marked." Secondly, a case, in which a woman died in labour, from rupture of the cervix uteri, there being no trace of a normal os uteri, save something like a dimple at its ordinary site. Thirdly, a case by professor Hamilton of Edinburgh, of a healthy young woman, who had menstruated regularly up to the time of her first pregnancy—who went to the full term—had severe labour pains, which were ineffectual from an adhesion of the vagina to the extent of an inch, about two inches from its external orifice, which was opened by an incision, when shortly after the labour terminated favourably.

We have thus given all of the important facts connected with this most interesting case.

### 7.—*On Incision in cases of Occlusion and Rigidity of the Uterus. By Samuel Ashwell, M. D.*

The object of this paper of Dr. Ashwell's is to show—

"1st. That incision is the safest remedy, where the os is in a state of firm and complete closure; or, in other words, where the uterus, so far as its lower orifice is concerned, is imperforate: and

2dly. That in examples of such extreme rigidity of the os, where, after hours of strong uterine effort, the power of dilatation is entirely absent, whether such rigidity arise from disease in the structural organization of the part, or has resulted from previous laceration and ulceration, incision is the best and safest treatment; far preferable to protracted and powerful dilatation of the os by the finger; or, on the principle of non-interference, to leaving the case to the natural efforts."

Complete closure of the os-uteri may result from adhesive inflammation, in cases where the aperture is unusually small, and as a consequence of "morbid deposit about the os and cervix, produced either by chronic inflammation or occurring as the result of former laceration or ulceration."

The only condition with which occlusion may be confounded, is obliquity of the uterus; particularly ante-version, in which the os is thrown far upwards and backwards; towards or at the promontory of the sacrum. In regard to the practicability and mode of distinguishing occlusion from obliquity, the Doctor observes—

"There can be but little difficulty in the diagnosis of instances of complete and firm closure of the os. When parturient effort is really established, the lower portion of the uterus, in the form of a tense and large globular mass, is generally forced down very low, sometimes so far, as nearly to reach the external entrance of the vagina. Thus a finger—at all practised in these inquiries—*must* detect an aperture, if there be one; and, if not, the spot where the os uteri, at the time of conception, had been.

A repetition of uterine action will afford abundant opportunities for careful re-examination; so that no apology for indiscreet and dangerous delay can exist. If, too, a spot shall be discovered—more depressed, and of different structure to the surrounding parts, indicating the site of the os uteri at the time of impregnation, it is impossible then to doubt about tne nature of the case; and the only question remaining to be determined, is the precise method of relief."

When upon the subject of treatment, and after urging in strong terms, the impropriety in these cases of occlusion of venesection, and permitting the labor to be protracted to a great length of time, in hopes of discovering an os uteri, or that nature will remedy the evil, the Doctor says: "there are two methods of remedying the closure of this important orifice:

1. By such an amount of pressure by the finger, female catheter, sound or bougie, as shall puncture or open the occlusion: and
2. By incision made by a bistoury or knife."

The first named method may be employed when the occlusion is slight, and depending upon a thin membrane stretched across the os. But the author differs from some writers upon the subject, in thinking that incision is preferable, when this membrane has become completely organized, firm and unyielding. And he also recommends incision when the occlusion is dependent upon adhesion or morbid deposit. He assigns two reasons for this preference. First, that if the finger or bougie, &c., be employed, contusion of the part will necessarily follow, and thus render the patient liable to local or general uterine inflammation. Secondly, he thinks that there is less liability to extensive laceration where a free incision is made, than where a small puncture is made by any of the above mentioned methods. In support of this last position, he cites some cases in which incision was employed, with the effect first named.

Rigidity, which is treated of in the second division of his subject, and which is of much more frequent occurrence than occlusion, may (in that form of it which requires incision) result from a contracted orifice being surrounded by a "structure almost entirely undilatable," or from the cicatrization of abscesses, ulcerations, &c., or from a hard tumor or malignant deposit, which alters the structure of the part.

If the nature of the case be made out in the early stage of labor, it is considered not only prudent but highly important to resort to bloodletting, antimony, opium, &c., with the hope of producing relaxation. But when these means have been resorted to and a sufficient time has been allowed to give nature, with these adjuvants, an opportunity of overcoming the rigidity, and without success, the author says: "The case may then be treated by artificial dilatation or by incision, or it may be left to nature." To adopt the last course, he considers, would be to consign the woman to unlimited laceration or death by inflammation from protracted uterine contraction. To artificial dilatation he is decidedly opposed, for the same reasons which have been assigned against its employment in occlusion. In regard to incision he thinks it should be at once resorted to, wnen it is perceived that nature is unequal to the task. "If there be distressing and constant pain about the neck or body of the uterus, or in any other part; if the countenance becomes turgid and dark; if perspiration issues at every pore, and the pulse is full, strong, quick and incompressible; and if these symptoms continue, although perhaps somewhat lessened by bleeding and antimony; there can be no doubt that recourse snould be had to the incision." We should not wait until the woman is worn out by fruitless contractions, and is already in u state of collapse, when the operation would of course be unavailing towards saving the life of the patient.

After describing the manner of performing the operation, (which is so simple, and will suggest itself so readily, as to render a description of it unnecessary here) Dr. Ashwell devotes the balance of his paper to detailing some cases in which incision had been employed, in which no unpleasant consequences grew out of its use, and success attended it when early enough resorted to.

